# The Association between Physical Activity and Cognitive Function: Data from the China Health and Nutrition Survey

**DOI:** 10.1155/2022/3438078

**Published:** 2022-06-20

**Authors:** Qiankun Huang, Jing Zhao, Weiqing Jiang, Wenfeng Wang

**Affiliations:** ^1^Minhang District Mental Health Center of Shanghai, Shanghai 201112, China; ^2^Shanghai Mental Health Center, Shanghai Jiao Tong University School of Medicine, Shanghai 201108, China; ^3^School of Science, Shanghai Institute of Technology, Shanghai 201418, China

## Abstract

**Background:**

Decreased cognitive function is a common problem in the old adults, which has high risk of progression to Alzheimer's diseases (AD) and other dementias. This study was aimed at finding out the association between physical activity and cognitive function.

**Methods:**

In total, 1514 participants with the age ≥ 55 years old registered in the China Health and Nutrition Survey (CHNS) database were selected in this study. The association between physical activity and cognitive function was analyzed via the generalized additive model. The association between the variables and the cognitive function score was expressed as *β* coefficient with 95% confidence intervals (CIs).

**Results:**

After adjusting age, ethnicity, stratum, marital status, education, memory status, and memory changes, the cognitive function score was increased by 0.011 points for every 1-point increase in domestic score (*β* = 0.011, *P* = 0.043). Subgroup analysis indicated that in the female group, for every 1-point increase in the domestic score, the cognitive function score increased by 0.019 points (*β* = 0.019, *P* = 0.017). In people with good memory status, each 1-point increase in domestic score increased the cognitive function score of 0.020 points (*β* = 0.020, *P* = 0.017).

**Conclusions:**

The decreased cognitive function was correlated with decreased domestic physical activity. The increased domestic physical activity was associated with an increased cognitive function in females and people with good memory status. The findings might offer a reference for deep understanding of the association between physical activity and cognitive function.

## 1. Introduction

Cognitive function includes memory, language, judgment, and attention, which may be impaired by neurodegeneration and vascular or dysthymia/dysphoria problems [1, 2]. Cognitive impairment is a common problem in the old adults, ranging from mild cognitive impairment (MCI) to dementia [3]. Cognitive impairment has attracted particular attention on account of its prevalence and clinical impact on patients [4]. Previous studies have reported that individuals with MCI have high risk of progression to Alzheimer's diseases (AD) and other dementias [5]. A study showed that the MCI conversion rate to dementia is about 10% per year, which is increased to 80%-90% after approximately 6 years [6]. This demonstrated that it is valuable to identify methods to prevent the deterioration of MCI to dementia. Currently, the effect of pharmacological treatment for mild cognitive impairment is modest or some even have no effect, and new therapeutic methods are urgently required for mild cognitive impairment [7]. Recently, nonpharmacological treatments attract more attention concerning the prevention of cognitive function decline.

Physical activity is a widely proposed nonpharmacological method for MCI therapy, and it is mainly composed of occupational, domestic, transportation, and leisure time activities [8]. Growing studies have demonstrated that physical activity can prevent the decline of cognitive function, and the global cognitive function has association with the amount of physical activity [9, 10]. Barnes et al. have demonstrated that physical activity increasing by 10% could significantly decrease the risk of MCI to dementia [11]. Another study also reported that low physical activity increased risk of MCI to dementia [12]. Currently, the investigation on the association of cognitive function and physical activity among Chinese population remains largely unclear. In this study, the association between physical activity and cognitive function was analyzed according to the data from the China Health and Nutrition Survey (CHNS) based on the generalized additive model. The results of this study might provide a reference for identifying the association between different physical activities and cognitive function and encourage the older adults to take part in more physical activities.

## 2. Methods

### 2.1. Study Population

The CHNS is a freely available database established in 1989. It is a large-scale and household-based cohort study with over 30,000 individuals in 9 provinces in China on the health conditions, nutritional statuses, and diseases of subjects by the Chinese Center for Disease Control and Prevention [13]. In the survey, the samples were selected via the random-cluster sampling process in a large higher-income city, a lower-income city, and four counties (1 high-, 2 middle-, and 1 low-income according to per-capita income provided by the National Bureau of Statics) within each province. Urban and suburban neighborhoods within the cities and villages and townships within the counties were selected randomly as the primary sampling unit, in which twenty households were randomly selected and all household members were interviewed [14]. The survey got the approval from the institutional review committees of the University of North Carolina, the Chinese Institute of Nutrition and Food Safety, and the China Center for Disease Control and Prevention. All participants provided the informed consents. Due to public availability of CNHS database, with private information of all patients being anonymized, the local ethics committee's approval was not required in our study. This cross-sectional study collected the data of 180,713 participants from the CHNS database. Among them, 15,923 were from 1989, 16,033 were from 1991, 15,058 were from 1993, 15,822 were from 1997, 17,054 were from 2000, 16,126 were from 2004, 18,785 were from 2006, 18,913 were from 2009, 23,060 were from 2011, and 23,939 were from 2015. The data of the participants in previous years were excluded (*n* = 139,781) including 15,602 from 1989, 15,070 from 1991, 13,354 from 1993, 14,365 from 1997, 15,359 from 2000, 14,946 from 2004, 16,358 from 2006, 17,112 from 2009, and 17,615 from 2011, and we only analyzed the data collected from the latest year. Participants who did not survey the data of cognitive function or did not complete the items of questionnaire concerning cognitive function were also excluded (*n* = 37,238). After exclusion of participants without the data on age, gender, height, weight, nationality, systolic blood pressure (SBP), diastolic blood pressure (DBP), sleep time, and memory changes (*n* = 2180), finally, 1514 subjects were included in the study. Among them, 23 people were from 2004, 41 people were from 2006, and 1450 were from 2015. Sensitive analysis depicted that no statistical difference was found concerning the demographic data and physical activity scores including cognitive function, domestic score, occupational score, transportation score, and leisure time score between participants completing the survey on cognitive function and participants finally included (Supplementary Table 1).

### 2.2. Data Extraction

Clinical data of 1514 subjects were collected including age, gender, nationality (Han or others), stratum (city, suburban, town or county capital city, or rural village), SBP (mmHg), DBP (mmHg), hypertension, diabetes, body mass index (BMI) (kg/m^2^), household income (yuan), marital status (never married, married, divorced, widowed, or separated), education, sleep time, history of smoking, drinking, drinking frequency, memory status (bad, good, or OK), memory changes (deteriorated, improved, or stayed the same), domestic score, occupational score, transportation score, and leisure time score.

### 2.3. Definition and Assessment of the Variables

The cognitive function score is divided into four parts including the immediate memory (10 scores), the delayed recall of a 10-word list (10 scores), counting backward from 20 (2 scores), and serial 7 subtraction (5 scores) [15]. The sum of the four scores makes the final score. The total global cognitive score ranged from 0 to 27. Higher scores indicate better cognitive function. The cognitive function test started with the immediate recall of a 10-word list. The interviewer (trained health worker) read ten words at a speed of two seconds per word. The participants were given two minutes to memorize the ten words. For each correct recalled word, a score of 1 was given. The participants were then asked to count back from 20 to 1. If the participants made a mistake in the first try, a second chance was given. A score of 2 was given to those answered correctly in the first try or 1 in the second try. After the count test, the participants were asked to do five consecutive subtractions of 7 from 100. Each correct subtraction was given a score of 1. Finally, the participants were asked to recall the 10-word list tested before. Each recalled word was given a score of 1.

The physical activity-related variables were calculated into four dimensions: domestic, occupational, transportation, and leisure time [8]. In our study, the data on these four dimensions were collected by staff-administered questionnaires [16]. The assessment of these four dimensions was based on metabolic equivalent- (MET-) hours-per-week to account for both intensity and time spent on activities [17]. Physical activities were reported in average hours-per-week spent in the past year. The level of physical activity was the product of time spent in each activity multiplied by specific MET values based on the “Compendium of Physical Activities” [18]. Domestic activity was measured based on four activities. The MET values assigned were as follows: 2.3 for buying food, 2.25 for preparing food or cooking, 2.15 for laundry, and 3.0 for sweeping rooms. Occupational activity was measured according to the intensity of specific jobs (light, moderate, and heavy), with assigned values of 2.0, 4.0, and 6.0, respectively. Evaluation of transportation activity was based on four commuting types to and from work or school. The MET values assigned were as follows: 3.0 for walking, 4.0 for bicycling, and 1.5 for motorized vehicle. The MET values used for leisure time activities were 4.5 for martial arts, 5.0 for gymnastics, dancing, or acrobatics, 7.5 for jogging or swimming, 6.0 for playing soccer, basketball, or tennis, and 5.0 for playing badminton, volleyball, or ping-pong.

Memory status of participants were defined based on the question “how is your memory: (1) very good; (2) good; (3) OK; (4) bad; (5) very bad.” Those who reported “bad” or “very bad” were defined as having a bad memory. Those who reported “very good” and “good” were combined into the “good” group. Memory change was assessed by the question “in the past twelve months, how has your memory changed: (1) improved; (2) stayed the same; (3) deteriorated.”

### 2.4. The Generalized Additive Model

**Figure 1:**
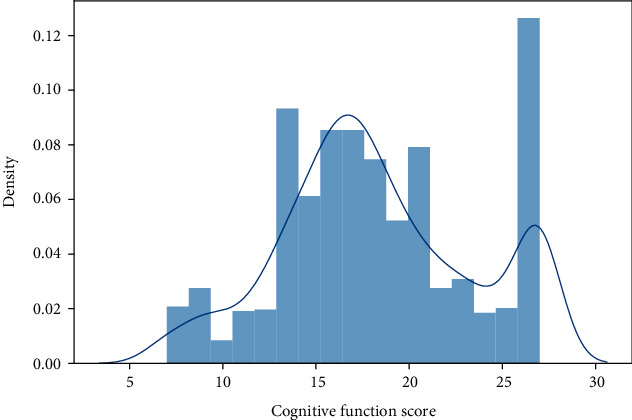
The histogram of cognitive function scores.

**Figure 2:**
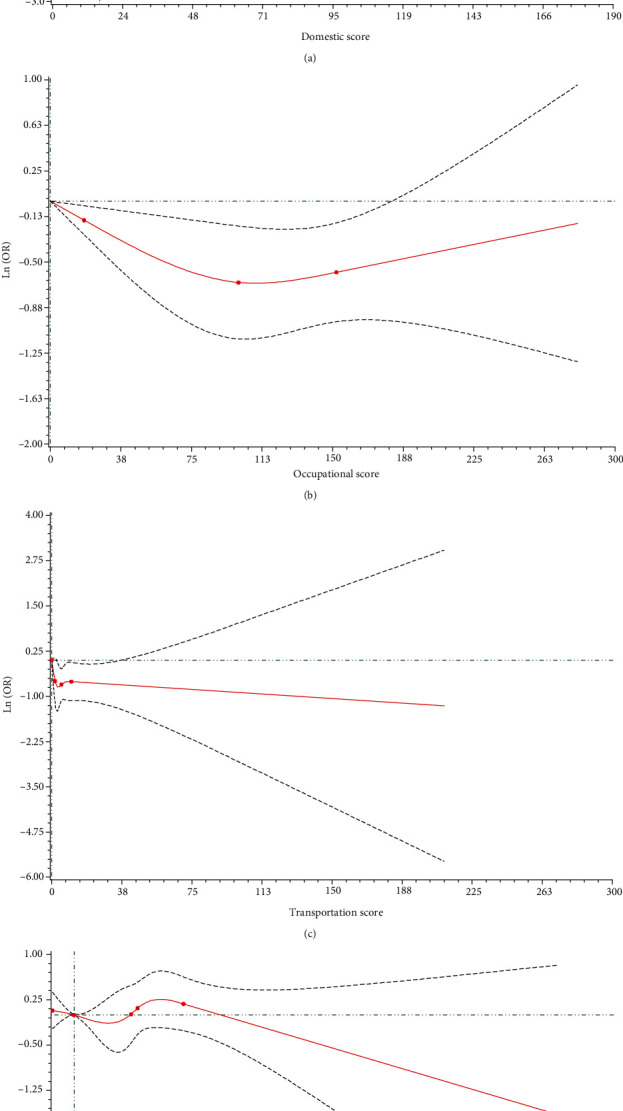
The RCS results between scores of (a) domestic, (b) occupational, (c) transportation, and (d) leisure time activities and cognitive function score.

**Figure 3:**
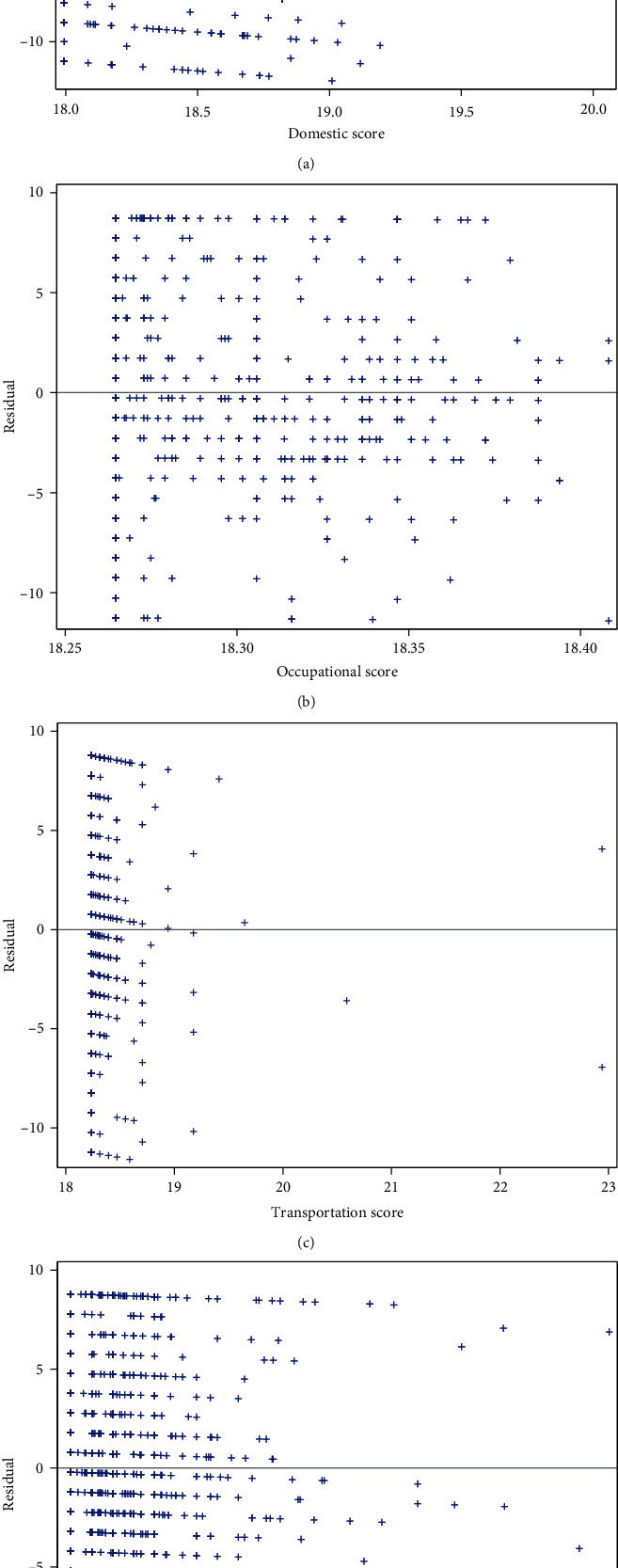
The residual graph of linear fitting between scores of (a) domestic, (b) occupational, (c) transportation, and (d) leisure time activities and cognitive function score.

In the present study, the cognitive function score data were seriously skewed (Figure 1). Then, the restricted cubic spline (RCS) was plotted and revealed that the association between physical activity and cognitive function was nonlinear (Figures 2(a)–2(d)). Additionally, the linear fitting was poor according to the linear fitting residual graph of cognitive function score and exercise-related score (Figures 3(a)–3(d)), so we chose the generalized additive model for analyzing the association between physical activity and cognitive function. The generalized additive model is an extension of generalized linear models that allows nonlinear functional forms between independent variables and the response [19]. The generalized additive model helps in improving the predictive accuracy of *Y*.

### 2.5. Statistical Analysis

Statistical analyses were performed by the use of SAS (9.4 Edition, SAS Institute, Cary, NC, USA). The measurement data were displayed as mean ± standard deviation (mean ± SD), and the enumeration data were presented as cases and frequency [*N* (%)]. The association between physical activity and cognitive function was analyzed via the generalized additive model. The association between the variables and the cognitive function score was expressed as *β* coefficient with 95% confidence intervals (CIs). All statistical analyses were bilateral, and *P* < 0.05 was considered statistically significant.

## 3. Results

### 3.1. The Characteristics of Subjects

**Figure 4:**
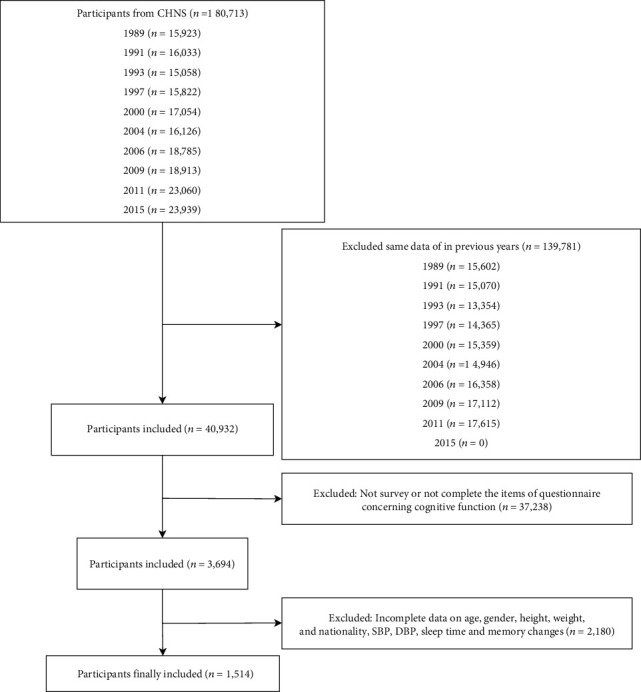
The screening process of the participants.

A total of 1514 participants were included and analyzed with the screening process shown in Figure 4. Among them, 943 were male, accounting for 62.29%, and 571 were female, accounting for 37.71%. Among the ethnic groups, 1427 were Han, accounting for 94.25%, and 87 were other ethnic groups, accounting for 5.75%. The average SBP was 135.03 mmHg ± 17.37 mmHg. The average DBP was 83.24 mmHg ± 10.44 mmHg. The mean BMI was 24.49 kg/m^2^ ± 3.42 kg/m^2^. In terms of education level, 861 people received junior middle school education or below, accounting for 56.87%, 490 people received high school education, accounting for 32.36%, and 163 people received university education or above, accounting for 10.77%. The average length of sleep was 7.61 h ± 1.22 h. Other baseline data are displayed in Table 1.

### 3.2. The Association between Physical Activity and Cognitive Functioning

According to the results in the univariate analysis, age, ethnics, marital status, education memory status, and memory changes were confounders of the association between physical activity and cognitive functioning. In the multivariate analysis, these confounders were adjusted to evaluate the association of physical activity including domestic, occupational, transportation, and leisure time scores with the cognitive function score. The data showed that for every 1-point increase in domestic score, the cognitive function score was increased by 0.011 points (*β* = 0.011, 95% CI: 0.001-0.022, *P* = 0.043) (Table 2).

### 3.3. Subgroup Analysis of Factors Influencing Cognitive Functioning

The detailed information of the domestic score, occupational score, transportation score, and leisure time score in females and males is shown in Supplementary Table 2. The mean domestic score was 12.45 in males and 45.69 in females. The mean occupational score was 31.06 in males and 7.63 in females. The mean transportation score was 2.27 in males and 1.03 in females. The mean leisure time was 17.44 in males and 23.63 in females. As for the domestic score, occupational score, transportation score, and leisure time score in people with different memory status, the mean domestic score was 25.89 in people with bad memory status, 25.31 in people with good memory status, and 24.33 in people with OK memory status. The mean occupational score was 8.22 in people with bad memory, 26.36 in people with good memory status, and 21.79 in people with OK memory status. The mean transportation score was 0.52 in people with bad memory status, 2.34 in people with good memory status, and 1.59 in people with OK memory status. The mean leisure time score was 18.20 in people with bad memory status, 20.50 in people with good memory status, and 19.43 in people with OK memory status.

As for subgroup analysis of different genders, there was no statistical significance in the four dimensions in males (all *P* > 0.05). In females, a 1-point increase in domestic score was associated with an increase of cognitive function score of 0.019 points (*β* = 0.019, 95% CI: 0.003-0.035, *P* = 0.017) (Table 3). An increased domestic score was associated with an elevated cognitive function in people with good memory status (*β* = 0.020, 95% CI: 0.004-0.036, *P* = 0.017) (Table 4).

## 4. Discussion

This study collected the clinical data of 1514 participants from the CHNS database and analyzed the association between physical activity-related four dimensions (occupational, domestic, transportation, and leisure time activities) and cognitive function based on the generalized additive model. The results indicated that the cognitive function score was increased by 0.011 points for every 1-point increase in domestic score after adjusting the significant variables in univariate analysis including age, ethnicity, stratum, marital status, education, memory status, and memory changes. In addition, subgroup analysis revealed that domestic score was a protective score for the cognitive function in females and people with good memory status. The findings might offer a reference for deep understanding of the association between cognitive function and physical activity.

Physical activity refers to the bodily movement which resulted from skeletal muscles that increases the consumption of energy [20]. The Australian Physical Activity and Sedentary Behavior Guidelines recommend adults ≥ 65 years to perform moderate-intensity physical activity for more than 30 min on most days of the week in addition to usual activities of daily living [21]. Physical activity was reported to have the ability to delay the decline of cognitive function and reduce the risk of dementia through improving blood supply to the brain and reinforcing the nervous system [22, 23]. Physical activity can also strengthen the cardiovascular, immune, and metabolic system functions and regulate the internal environment of the brain [24]. According to the Copenhagen Consensus statement 2019, physical activity was confirmed to be effective in improving the cognitive and brain health in older adults [25]. Physical activity has different domains including occupational, domestic, transportation, and leisure time activities. Previously, studies have revealed that some domestic physical activities including sweeping, vacuuming, window cleaning, and lawn mowing are moderate intensity for middle-aged or older people [26]. In the present study, domestic activity was identified to be associated with the cognitive function in females, and females tended to do more domestic activities than males. The increased domestic score was positively correlated with the cognitive function score. Previously, a study has shown that domestic activity was beneficial for human health [27]. According to the data of a cross-sectional study with 1070 participants in Bahia, domestic physical activities have a positive impact on the control of high blood pressure [28]. This evidence supported the conclusion of our study. Domestic physical activity was also observed to have gender-specific effects on health indicators in Europe [27]; this may be due to the fact that females tend to have more domestic physical activity than males.

Herein, we found elevated domestic score was associated with increased cognitive function in people with good memory status. Memory is fundamental in our day-to-day lives, and memory status may influence the everyday function of people [29]. For those with good memory status, domestic activities such as buying food, preparing food or cooking, laundry, or sweeping rooms were encouraged. The reason may be that people with good memory status may finish the domestic activities such as buying food, preparing food or cooking, laundry, or sweeping rooms more precisely and completely than people with OK or bad memory status. Although the mean and median domestic score were similar in people with bad, OK, or good memory, the domestic activity intensity in those with good memory status might be higher than that in people with OK or bad memory status. In this study, the maximum domestic score in people with good memory was higher than in those with bad or OK memory, which indicated that people with good memory may be engaged in more and longer time of domestic activities than people with OK or bad memory status. Older adults who have moderate or poor memory may be more sensitive to any negative function changes in daily activities and they may feel that they have less control over their cognitive performance, which lead them to avoid doing activities including domestic activities [30]. Additionally, people with good memory can do domestic activities such as buying food, which help them involve more social networks through communicating with others and then help preserve the cognitive function [31].

In the present study, occupational activity was not statistically associated with the cognitive function in older people; this may be because the CNHS database collected the data of individuals aged 45 and above [32]. In our study, we included the participants with the age ≥ 55 years old and most of them may be retired and those people were not engaged in the occupational activities. Thus, the occupational activities might have little association with the cognitive function in those people. In this study, no statistical association was found between transportation activity and cognitive function. For many older people, walking was the major transportation activity for them, and other transportation activities such as bicycling and motorized vehicle were not suitable for them. Additionally, for older people, they like to stay at home or just walk at the place close to home. They just want to stay in the place they are familiar with and do not like to go far away. The walking intensity was small in these people. For people that need to go far away, the free older persons' bus pass encourages them to choose public transports rather than cycling or motorized vehicle [33]. Leisure time activity was found not associated with cognitive function in older adults in the present study. As our data was collected from 1989 to 2015, leisure time activity was not popular in older people during that time.

The strength of this study was that we deeply analyzed the association between physical activity and cognitive function from four domains of physical activity including occupational, domestic, transportation, and leisure time activities especially in the Chinese population. In addition, subgroup analysis was performed in terms of different genders and people with different memory status, which might more clearly stratified the association between physical activity and cognitive function in different kinds of people. There were some limitations of this study. Firstly, there was no uniform tools for measuring the intensity of physical activity till now, which may have an impact on the results of this study. Secondly, some data were collected via questionnaire and self-reported, which may result in an uncontrollable source of bias. Thirdly, the CHNS database covers 228 communities in Heilongjiang, Liaoning, Shandong, Henan, Jiangsu, Hunan, Hubei, Guangxi, and Guizhou provinces, which cannot represent the whole country of China, indicating that the results of our study should be interpreted with caution. Fourthly, this was a cross-sectional study; we only found the association between physical activity and cognitive function, but could not identify the cause-effect between them. In the future, prospective randomized controlled trials (RCTs) or longitudinal cohort studies were required to verify the findings in this study.

## 5. Conclusions

In the study, the clinical data of 1514 subjects from the CHNS database were analyzed to explore the association between physical activity and cognitive function. The results indicated that decreased cognitive function was correlated with the reduction of domestic score after adjusting age, ethnicity, stratum, marital status, education, memory status, and memory changes especially in females and people with good memory status. The results of our study might offer a reference for deep understanding of the association between special physical activity and cognitive function in older people.

## Figures and Tables

**Table 1:** The characteristics of the participates.

Variable	Description
Age	64.20 ± 6.94
Gender	
Male	943 (62.29)
Female	571 (37.71)
Ethnicity	
Han	1427 (94.25)
Others	87 (5.75)
Stratum, *n* (%)	
City	608 (40.16)
Suburban	223 (14.73)
Town or county capital city	256 (16.91)
Rural village	427 (28.20)
Hypertension, *n* (%)	
No	16 (8.56)
Yes	171 (91.44)
Diabetes	
No	671 (90.55)
Yes	70 (9.45)
Household income (yuan)	20000 (5000, 60000)
SBP	135.03 ± 17.37
DBP	83.24 ± 10.44
BMI	24.49 ± 3.42
Marital status	
Married	1387 (91.61)
Widowed	109 (7.20)
Separated	1 (0.07)
Never married	3 (0.20)
Divorced	14 (0.92)
Education	
Middle school or below	861 (56.87)
High school	490 (32.36)
University and above	163 (10.77)
Sleep time	7.61 ± 1.22
Smoke	
No	1145 (75.63)
Yes	369 (24.37)
Drink	
No	1079 (71.27)
Yes	435 (28.73)
Drink frequency	
No drink	1079 (71.27)
Less than once a week	121 (7.99)
1-2 times a week	87 (5.75)
3-4 times a week	48 (3.17)
Every day	179 (11.82)
Memory status	
Good	708 (46.76)
OK	616 (40.69)
Bad	190 (12.55)
Memory changes	
Improved	30 (1.98)
Stayed the same	924 (61.03)
Deteriorated	560 (36.99)
Physical activities	
Domestic score	24.99 ± 28.31
Occupational score	22.22 ± 51.32
Transportation score	1.80 ± 9.44
Leisure time score	19.78 ± 28.36

SBP: systolic blood pressure; DBP: diastolic blood pressure; BMI: body mass index. The minimum domestic score was 0 and the maximum domestic score was 182.70. The minimum occupational score was 0 and the maximum occupational score was 280.00. The minimum transportation score was 0 and the maximum occupational score was 210.00. The minimum leisure time score was 0 and the maximum occupational score was 270.00.

**Table 2:** Univariate and multivariate analysis of factors influencing cognitive functioning.

Physical activity score	Univariate analysis		Multivariate analysis^∗^	
	*β* (95% CI)	*P*	*β* (95% CI)	*P*
Domestic score	0.011 (0.002-0.021)	**0.017**	0.011 (0.001-0.022)	**0.043**
Occupational score	0.001 (-0.005-0.006)	0.844	-0.002 (-0.008-0.003)	0.403
Transportation score	0.022 (-0.005-0.050)	0.114	0.012 (-0.014-0.039)	0.369
Leisure time score	0.003 (-0.006-0.013)	0.473	-0.003 (0.012-0.006)	0.510

^∗^Multivariable analysis adjusting age, ethnicity, stratum, marital status, education, memory status, and memory changes.

**Table 3:** The analysis of subgroup considering different genders.

Physical activity score	Males		Females	
	*β* (95% CI)	*P*	*β* (95% CI)	*P*
Domestic score	0.001 (-0.015-0.017)	0.885	0.019 (0.003-0.035)	**0.017**
Occupational score	-0.002 (-0.008-0.003)	0.439	-0.004 (-0.020-0.011)	0.595
Transportation score	-0.002 (-0.035-0.032)	0.925	0.035 (-0.009-0.079)	0.122
Leisure time score	-0.007 (-0.019-0.005)	0.269	0.002 (-0.012-0.016)	0.824

Multivariable analysis adjusting age, ethnicity, stratum, marital status, education, memory status, and memory changes.

**Table 4:** The association between physical activities and cognitive function in people with different memory status.

Physical activity	Good		Bad		OK	
	*β* (95% CI)	*P*	*β* (95% CI)	*P*	*β* (95% CI)	*P*
Domestic score	0.020 (0.004-0.036)	**0.017**	0.025 (-0.001-0.050)	0.059	-0.007 (-0.024-0.011)	0.456
Occupational score	-0.002 (-0.010-0.005)	0.498	0.011 (-0.013-0.035)	0.360	-0.004 (-0.011-0.004)	0.366
Transportation score	0.020 (-0.012-0.052)	0.215	0.062 (-0.178-0.301)	0.616	-0.017 (-0.076-0.041)	0.558
Leisure time score	-0.002 (-0.015-0.011)	0.753	0.011 (-0.016-0.038)	0.426	-0.009 (-0.024-0.007)	0.283

Multivariable analysis adjusting age, ethnicity, stratum, marital status, education, and memory changes.

## Data Availability

The data utilized to support the findings are available from the corresponding authors upon request.

## Supplementary Materials

Supplementary Materials Sensitivity analysis of the missing data and the detailed information of physical activity scores in people from subgroups.
