# Supplementary material for: The Association between Physical Activity and Cognitive Function: Data from the China Health and Nutrition Survey
Source: Behav Neurol. 2022 Jun 20;2022:3438078. doi: 10.1155/2022/3438078 (PMC9236826; doi:10.1155/2022/3438078)
Supplement: Supplementary Materials — Sensitivity analysis of the missing data and the detailed information of physical activity scores in people from subgroups. [file 3438078.f1.docx]

Supplementary Table 1 Sensitivity analysis of the data between participants with incomplete data and participants finally included

| Variable | Before (n=3694) | After (n=1514) | Statistical magnitude | *P* |
| --- | --- | --- | --- | --- |
| Age, Mean ± SD | 64.56 ± 7.56 | 64.20 ± 6.94 | t=1.597 | 0.110 |
| Gender n (%) |  |  | χ^2^=2.156 | 0.142 |
| Male | 2220 (60.73) | 943 (62.29) |  |  |
| Female | 1474 (39.90) | 571 (37.71) |  |  |
| Ethnicity, n (%) |  |  | χ^2^=1.324 | 0.250 |
| Han | 2817 (93.37) | 1427 (94.25) |  |  |
| Others | 200 (6.63) | 87 (5.75) |  |  |
| Stratum, n (%) |  |  | χ^2^=0.427 | 0.935 |
| City | 1484 (40.17) | 608 (40.16) |  |  |
| Suburban | 546 (14.78) | 223 (14.73) |  |  |
| Town or county capital city | 600 (11.52) | 256 (16.91) |  |  |
| Rural village | 1064 (28.80) | 427 (28.20) |  |  |
| Hypertension, n (%) |  |  | χ^2^=0.003 | 0.960 |
| No | 320 (8.66) | 16 (8.56) |  |  |
| Yes | 3374 (91.34) | 171 (91.44) |  |  |
| Diabetes, n (%) |  |  | χ^2^=0.102 | 0.750 |
| No | 3340 (90.17) | 671 (90.55) |  |  |
| Yes | 364 (9.83) | 70 (9.45) |  |  |
| Household income, M (Q_1_, Q_3_) | 22000 (5000, 60000) | 20000 (5000, 60000) | Z=1.925 | 0.054 |
| SBP, Mean ± SD | 133.87 ± 20.56 | 135.03 ± 17.37 | t=1.898 | 0.058 |
| DBP, Mean ± SD | 84.16 ± 17.36 | 83.24 ± 10.44 | t=1.905 | 0.057 |
| BMI, Mean ± SD | 24.21 ± 5.56 | 24.49 ± 3.42 | t=1.785 | 0.074 |
| Marital status, n (%) |  |  |  |  |
| Married | 2745 (91.38) | 1387 (91.61) |  |  |
| Widowed | 216 (7.19) | 109 (7.20) |  |  |
| Separated | 2 (0.07) | 1 (0.07) |  |  |
| Never married | 6 (0.20) | 3 (0.20) |  |  |
| Divorced | 35 (1.17) | 14 (0.92) |  |  |
| Education, n (%) |  |  | χ^2^=0.417 | 0.812 |
| Middle school or below | 1420 (56.66) | 861 (56.87) |  |  |
| High school | 800 (31.92) | 490 (32.36) |  |  |
| University and above | 286 (11.41) | 163 (10.77) |  |  |
| Sleep time | 7.68 ± 1.5 | 7.61 ± 1.22 |  |  |
| Smoke, n (%) |  |  | χ^2^=2.207 | 0.137 |
| No | 2362 (77.60) | 1145 (75.63) |  |  |
| Yes | 682 (22.40) | 369 (24.37) |  |  |
| Drink, n (%) |  |  | χ^2^=3.376 | 0.066 |
| No | 1600 (74.01) | 1079 (71.27) |  |  |
| Yes | 562 (25.99) | 435 (28.73) |  |  |
| Drink frequency, n (%) |  |  | χ^2^=0.107 | 0.999 |
| No drank | 1556 (71.97) | 1079 (71.27) |  |  |
| Less than once a week | 175 (8.09) | 121 (7.99) |  |  |
| 1-2 times a week | 119 (5.50) | 87 (5.75) |  |  |
| 3-4 times a week | 55 (2.54) | 48 (3.17) |  |  |
| Every day | 257 (11.89) | 179 (11.82) |  |  |
| Memory status, n (%) |  |  | χ^2^=0.815 | 0.665 |
| Good | 1122 (47.60) | 708 (46.76) |  |  |
| OK | 926 (39.29) | 616 (40.69) |  |  |
| Bad | 309 (13.11) | 190 (12.55) |  |  |
| Memory changes, n (%) |  |  | χ^2^=0.476 | 0.788 |
| Improved | 75 (2.03) | 30 (1.98) |  |  |
| Stayed the same | 2290 (61.99) | 924 (61.03) |  |  |
| Deteriorated | 1329 (35.98) | 560 (36.99) |  |  |
| Physical activity, M (Q_1_, Q_3_) |  |  | Z=1.217 | 0.224 |
| Domestic score | 19.37 (0.79, 39.14) | 15.93 (0, 43.20) | Z=0.590 | 0.555 |
| Occupational score | 0 (0, 0) | 0 (0, 0) | Z=0.570 | 0.569 |
| Transportation score | 0 (0, 0) | 0 (0, 0) | Z=0.302 | 0.762 |
| Leisure time score | 10 (0, 21) | 11.50 (0, 27.00) | Z=-0.241 | 0.810 |

SBP: systolic blood pressure, DBP: diastolic blood pressure, BMI: body mass index, SD: standard deviation

Supplementary Table 2 The detailed physical activity scores of people with different genders or memory status

| Variable | Total | Gender | |  | Memory status | | |
| --- | --- | --- | --- | --- | --- | --- | --- |
|  |  | Male | Female |  | Bad | Good | OK |
| Domestic score |  |  |  |  |  |  |  |
| Mean (SD) | 24.99 (28.31) | 12.45 (20.22) | 45.69 (27.62) |  | 25.89 (30.00) | 25.31 (29.06) | 24.33 (26.90) |
| Median | 15.93 | 0.00 | 44.45 |  | 16.02 | 15.93 | 15.93 |
| Q_1_, Q_3_ | 0.00, 43.20 | 0.00, 16.77 | 29.05, 60.55 |  | 0.00, 42.35 | 0.00, 44.83 | 0.00, 42.35 |
| Min, Max | 0.00, 182.70 | 0.00, 137.35 | 0.00, 182.70 |  | 0.00, 131.60 | 0.00, 182.70 | 0.00, 124.13 |
| Occupational score |  |  |  |  |  |  |  |
| Mean (SD) | 22.22 (51.32) | 31.06 (59.46) | 7.63 (28.37) |  | 8.22 (28.79) | 26.36 (54.72) | 21.79 (51.97) |
| Median | 0.00 | 0.00 | 0.00 |  | 0.00 | 0.00 | 0.00 |
| Q_1_, Q_3_ | 0.00, 0.00 | 0.00, 30.00 | 0.00, 0.00 |  | 0.00, 0.00 | 0.00, 16.00 | 0.00, 0.00 |
| Min, Max | 0.00, 280.00 | 0.00, 280.00 | 0.00, 182.67 |  | 0.00, 160.00 | 0.00, 280.00 | 0.00, 280.00 |
| Transportation score |  |  |  |  |  |  |  |
| Mean (SD) | 1.80 (9.44) | 2.27 (9.38) | 1.03 (9.49) |  | 0.52 (2.74) | 2.34 (12.24) | 1.59 (6.61) |
| Median | 0.00 | 0.00 | 0.00 |  | 0.00 | 0.00 | 0.00 |
| Q_1_, Q_3_ | 0.00, 0.00 | 0.00, 0.00 | 0.00, 0.00 |  | 0.00, 0.00 | 0.00, 0.00 | 0.00, 0.00 |
| Min, Max | 0.00, 210.00 | 0.00, 210.00 | 0.00, 210.00 |  | 0.00, 24.50 | 0.00, 210.00 | 0.00, 105.00 |
| Leisure time score |  |  |  |  |  |  |  |
| Mean (SD) | 19.78 (28.36) | 17.44 (26.59) | 23.63 (30.70) |  | 18.20 (24.89) | 20.50 (31.59) | 19.43 (25.29) |
| Median | 11.50 | 10.50 | 21.00 |  | 10.50 | 10.50 | 13.75 |
| Q_1_, Q_3_ | 0.00, 27.00 | 0.00, 21.00 | 0.00, 33.00 |  | 0.00, 27.00 | 0.00, 27.00 | 0.00, 25.50 |
| Min, Max | 0.00, 270.00 | 0.00, 270.00 | 0.00, 255.00 |  | 0.00, 156.00 | 0.00, 270.00 | 0.00, 217.50 |

SD: standard deviation
